# Alterations in DNA damage response and repair genes as potential biomarkers for immune checkpoint blockade in gastrointestinal cancer

**DOI:** 10.20892/j.issn.2095-3941.2020.0708

**Published:** 2021-09-28

**Authors:** Yujiao Wang, Xi Jiao, Shuang Li, Huan Chen, Xin Wei, Chang Liu, Jifang Gong, Xiaotian Zhang, Xicheng Wang, Zhi Peng, Changsong Qi, Zhenghang Wang, Yanni Wang, Na Zhuo, Jianling Zou, Henghui Zhang, Jian Li, Lin Shen, Zhihao Lu

**Affiliations:** 1 Department of Gastrointestinal Oncology, Key Laboratory of Carcinogenesis and Translational Research (Ministry of Education), Peking University Cancer Hospital & Institute, Beijing 100142, China; 2 Department of Gastric & Colorectal Surgery, The First Hospital of Jilin University, Changchun 130021, China; 3 Genecast Precision Medicine Technology Institute, Beijing 100192, China; 4 Life Sciences Institute, Zhejiang University, Hangzhou 310058, China; 5 Institute of Infectious Diseases, Beijing Ditan Hospital, Capital Medical University, Beijing 100015, China

**Keywords:** Gastrointestinal cancer, DDR gene mutation, immunotherapy, biomarker

## Abstract

**Objective:**

Immune checkpoint inhibitors (ICIs) have achieved remarkable results in cancer treatments. However, there is no effective predictive biomarker for gastrointestinal (GI) cancer.

**Methods:**

We conducted integrative analyses of the genomic and survival data of ICI-treated GI cancer patients from the Memorial Sloan Kettering Cancer Center cohort (MSK-GI, *n* = 227), the Janjigian cohort (*n* = 40), and the Peking University Cancer Hospital & Institute cohort (PUCH, *n* = 80) to determine the possible associations between DNA damage response and repair (DDR) gene mutations and clinical outcomes. Data from The Cancer Genome Atlas database were analyzed to determine the possible correlations between DDR gene mutations and the tumor microenvironment.

**Results:**

In the MSK cohort, the presence of ≥ 2 DDR gene mutations was correlated with prolonged overall survival (OS). The Janjigian and PUCH cohorts further confirmed that subgroups with ≥ 2 DDR gene mutations displayed a prolonged OS and a higher durable clinical benefit. Furthermore, the DDR gene mutation load could be considered as an independent prognostic factor, and exhibited a potential predictive value for survival in GI cancer patients treated with ICIs. Mechanistically, we showed that the presence of ≥ 2 DDR gene mutations was correlated with higher levels of tumor mutation burden, neoantigen, and T cell infiltration.

**Conclusions:**

The DDR gene mutation status was correlated with favorable clinical outcomes in GI cancer patients receiving ICIs, which could serve as a potential biomarker to guide patient selection for immunotherapy.

## Introduction

Gastrointestinal (GI) cancer ranks among the world’s most frequent cancer and accounts for a significant proportion of cancer-related deaths^1,2^. Immunotherapy represents a landmark therapeutic innovation in anticancer therapy, and has been approved in the treatment of various types of tumors, including GI cancer^3,4^. However, only a small proportion of patients with GI cancer benefit from immune checkpoint inhibitors (ICIs), because of a lack of optimal biomarkers^5^. To date, only high microsatellite instability (MSI-H) has been validated as a predictive biomarker in clinical trials^6^. Programmed death ligand-1 (PD-L1) shows a limited predictive value in colorectal cancer (CRC) and esophageal cancer (EC) due to challenges such as antibody uniformity and expression heterogeneity^7–12^. In addition, tumor mutation burden (TMB) is an important but controversial biomarker for GI cancer patients in different trials^13,14^. Therefore, it is imperative to develop additional biomarkers for immune checkpoint blockade in GI cancer to identify patients likely to respond to immunotherapy.

Previous studies have shown that the prominent role of DNA damage response and repair (DDR) alterations enhanced antitumor immunity through the production of neoantigens, favoring immune cell recruitment^15–17^. Classically, deficiency of DDR genes has been shown to guide clinical practice such as chemotherapy and radiotherapy in the treatment of cancer^16,18–21^. Emerging evidence has verified that DDR gene mutations have been correlated with prolonged survival in non-small cell lung cancer (NSCLC) and urethral carcinoma patients receiving PD(L)-1 antibodies^22,23^. However, a comprehensive knowledge of DDR gene mutations in GI cancer patients remains unclear and needs to be further investigated to guide ICI therapy.

In the current study, we collected next-generation sequencing data, evaluated the predictive value of DDR gene mutations in different cohorts, and elucidated the effects of DDR mutation status on the tumor microenvironment (TME). Our results may provide a biomarker to help predict the response and survival benefit from ICIs in GI cancer patients.

## Materials and methods

### Study design and patients

We utilized the genomic and clinical data of ICI-treated GI cancer patients from 3 independent clinical cohorts: (1) the Memorial Sloan Kettering Cancer Center (MSK) cohort^14^ (http://www.cbioportal.org/study?id=tmb_mskcc_2018) included 1,610 patients receiving ICIs in its pan-cancer analysis, and its GI subgroup contained 227 patients; (2) the Janjigian cohort (https://www.cbioportal.org/study/summary?id=egc_msk_2017) was comprised of 40 patients with esophagogastric cancer who received treatment with a programmed cell death-1 (PD-1) inhibitor alone or together with a cytotoxic T-lymphocyte-associated protein-4 (CTLA-4) inhibitor^24^; and (3) the Peking University Cancer Hospital & Institute (PUCH) cohort included 80 GI cancer patients who received ICI treatment from August 2015 to May 2019 (**Table 1**). Responses to immunotherapy were measured by a clinical radiographic assessment based on the Response Evaluation Criteria in Solid Tumors (RECIST) 1.1 and modified RECIST 1.1 for immune based therapeutics (iRECIST). Durable clinical benefit (DCB) was defined as complete response (CR), partial response (PR), or stable disease (SD) lasting ≥ 6 months; no durable benefit (NDB) was defined as progressive disease (PD) or SD lasting < 6 months after the beginning of treatment^25^. We also identified 92 stage II–III gastric cancer patients from the PUCH cohort who received primary gastric cancer resection and adjuvant platinum/5-fluorouracil-based chemotherapy (PUCH-ACT) as a nonimmunotherapy cohort to further validate the prognostic value of the DDR gene mutation load^26^.

**Table 1:** Clinical characteristics of patients from the 3 immunotherapeutic cohorts

Characteristics	MSK-GI cohort (*n* = 227)	Janjigian cohort (*n* = 40)	PUCH cohort (*n* = 80)
Age (years)			
≥ 65	74 (32.6%)	18 (45.0%)	22 (27.5%)
< 65	153 (67.4%)	22 (55.0%)	58 (72.5%)
Gender			
Male	152 (67.0%)	33 (82.5%)	56 (70.0%)
Female	75 (33.0%)	7 (17.5%)	24 (30.0%)
Tumor type			
Esophagogastric cancer	118 (52.0%)	40 (100.0%)	60 (75.0%)
Colorectal cancer	109 (48.0%)	0 (0%)	20 (25.0%)
Metastasis			
Yes	98 (43.2%)	27 (67.5%)	100 (100%)
No	129 (56.8%)	13 (32.5%)	0 (0%)
PD-L1			
Positive	NA	13 (32.5%)	29 (36.3%)
Negative		6 (15.0%)	27 (33.8%)
NA		21 (52.5%)	24 (30.0%)
MSI status			
MSI-H/dMMR	NA	5 (12.5%)^a^	22 (27.5%)
MSI-L/MSS/pMMR		35 (87.5%)	44 (55.0%)
NA		0 (0%)	14 (17.5%)
Drug type			
Anti-PD-1/PD-L1 therapy	185 (81.5%)	26 (65.0%)	69^b^ (86.3%)
Anti-CTLA-4 therapy	3 (1.3%)	0 (0%)	0 (0%)
Anti-PD-1/PD-L1+anti-CTLA-4 therapy	39 (17.2%)	14 (35.0%)	11 (13.8%)

Percentages might not total 100% because of rounding. NA, not available. ^a^MSI sensor method was used to evaluated MSI status in the Janjigian cohort. Samples with a score ≥ 10 were classified as MSI-H. ^b^Three patients received PD-1 inhibitor plus chemotherapy, and 1 patient received PD-1 inhibitor plus apatinib. MSI = microsatellite instability

For further analyses during this study, we obtained data from The Cancer Genome Atlas (TCGA) cohort of GI cancer (esophageal cancer, *n* = 184; gastric cancer, *n* = 439; colorectal cancer, *n* = 380) to determine the possible correlations between DDR mutation status and the tumor microenvironment.

### Targeted tumor next-generation sequencing

Tumors from the MSK-GI and Janjigian cohorts were analyzed using the Integrated Mutation Profiling of Actionable Cancer Targets (MSK-IMPACT) clinical sequencing assay, which is a next-generation sequencing platform based on hybridization capture^14,24^. Whole-exome sequencing (WES) of DNA was analyzed in tumors together with white blood cell samples of the patients in the PUCH cohort. The TMB was measured by analyzing somatic mutations per megabase (mutation/Mb). We selected a cutoff of the top 25% of the TMB as defining a tumor as TMB-High in each cohort (MSK-GI: 10.8 mutations/Mb; Janjigian cohort: 10 mutations/Mb; PUCH cohort: 10 mutations/Mb).

### Assessment of DDR mutation status

Based on PubMed searches and the National Center for Biotechnology Information Gene and BioSystems Databases, the MSK-IMPACT panel including a total of 34 genes was previously considered as DDR gene-related (**Supplementary Table S1**)^21,23^. A DDR gene list assembled using 6 major DDR pathways was defined as shown in **Supplementary Table S1**^21,23^. All loss-of-function alterations were classified as deleterious, such as nonsense mutations, frameshift mutations, or splice site alterations.

### Detection of PD-L1 expression

In the PUCH cohort, PD-L1 expression was detected by immunohistochemical staining of FFPE sections using an anti-PD-L1 antibody (rabbit, clone SP142, 1:100; Spring Bioscience, Pleasanton, CA, USA). PD-L1 positivity was defined as a staining cell percentage ≥ 1% of tumor and immune cells.

### Correlations between DDR gene mutations and the TME

We obtained genomic and mRNA data on GI cancer from TCGA on the website (https://gdc.cancer.gov/about-data/publications/pancanatlas). The expression data for mRNA in RNA-Seq by expectation-maximization (RSEM) values were transformed into log_10_(RSEM + 1). Previously published immune-related signatures were used to characterize the tumor immune microenvironment (**Supplementary Table S2**). We calculated the signature scores of patients by averaging of the included genes in the corresponding signature gene sets. To quantify the infiltration of immune cells in the TME, we used single-sample gene set enrichment analysis (ssGSEA) by the Gene Set Variation Analysis package to predict the distributions of various types of immune cells in tumors^27,28^. In addition, we obtained the tumor neoantigens of GI cancer patients from TCGA cohort data directly using the methods based on a previous study^29^. The mutation was possibly considered antigenic if it was predicted to generate a neopeptide with affinity less than 500 nM, and its corresponding gene expressed more than 10 transcripts per million.

### Statistical analysis

R statistical software, version 3.6.1 (The R Project for Statistical Computing, Vienna, Austria) and SPSS statistical software for Windows, version 23.0 (SPSS, Chicago, IL, USA) were used for the analyses. Categorical data were analyzed by the chi-squared test or Fisher’s exact test, as appropriate. Kaplan-Meier curves were used to determine the survival outcomes including overall survival (OS) and progression-free survival (PFS) estimations. Time-dependent receiver operating characteristic (ROC) curve analysis was used to assess the predictive accuracy of the DDR and other potential biomarkers for immunotherapy. Univariate Cox regression analysis was conducted to evaluate the prognostic value of current biomarkers for patient survival. Multivariate Cox regression analysis was conducted to determine the independent prognostic biomarkers of OS. Student’s *t-*test was used to determine the difference between 2 groups; and nonparametric tests were used when data were not normally distributed. *P*-values < 0.05 were assumed to be statistically significant.

## Results

### Patient characteristics

This study was based on 3 cohorts of GI cancer patients receiving ICI treatment. The MSK-GI cohort is a publicly available dataset consisting of 227 patients with esophagogastric cancer (*n* = 118) and colorectal cancer (*n* = 109). The median age of the cohort was 59-years-old (ranging from 19–87 years) with the majority being males (67.0%), which is reflective of GI cancer patients. The other data from the Janjigian and PUCH cohorts included 40 and 80 patients, respectively. **Table 1** provides a summary of the patient characteristics from these 3 immunotherapeutic cohorts.

### Association between DDR mutation status and GI cancer prognosis in the MSK cohort

**Figure 1:**
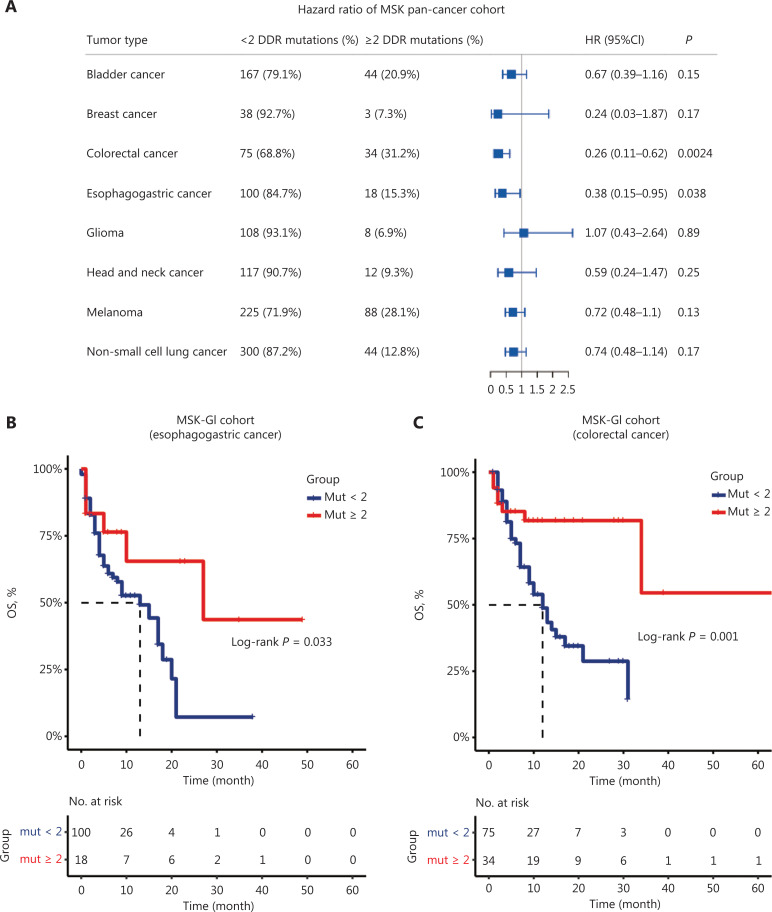
The association between DNA damage response and repair (DDR) gene mutations and survival outcomes of the Memorial Sloan Kettering Cancer Center (MSK) cohort. (A) Forest plot of hazard ratios (univariate analysis) describing the association between the number of DDR gene mutations (≥ 2 *vs.* < 2) and overall survival (OS) in pan-cancer from the MSK cohort. (B, C) Kaplan-Meier survival curves of overall survival comparing ≥ 2 DDR gene mutations with < 2 DDR gene mutations in esophagogastric cancer (*n* = 118) and colorectal cancer (*n* = 109). GI cancer, gastrointestinal cancer; Mut, mutation; HR, hazard ratio; CI, confidence interval.

We first analyzed the MSK pan-cancer cohort, consisting of 1,610 primary tumors assayed by the MSK-IMPACT sequencing panel to assess the prognostic value of DDR gene mutations. We found that the presence of DDR gene mutations predicted clinical survival in pan-cancer, and noticed that only when setting the DDR gene mutation number as 2, the prognostic value of the DDR gene mutation load was statistically significant in both esophagogastric cancer and the CRC subgroups (**Figure 1A and Supplementary Figure S1**). We next performed time-dependent ROC analysis to consider the number of DDR gene mutations as a continuous variable, and the highest value of the Youden index was for a cutoff value of 2 (**Supplementary Figure S2**). We therefore set DDR gene mutations = 2 as a cutoff value in our subsequent studies. **Figure 1B and 1C** shows that patients with ≥ 2 DDR gene mutations had improved OS in both esophagogastric cancer (*n* = 118, median OS: 27 *vs.* 13 months, *P* = 0.033) and colorectal cancer [*n* = 109, median OS: not reached (NR) *vs.* 12 months, *P* = 0.001] (**Figure 1B and 1C**). Notably, different DDR pathways may have distinct prognostic values for immunotherapy survival in the MSK cohort (**Supplementary Figure S3**). The mutations of genes involved in mismatch repair (MMR), nucleotide excision repair (NER), Fanconi anemia (FA), and homologous recombination (HR) were associated with a favorable prognosis, but mutations in the Checkpoint pathway did not show these relationships. The number and frequency of DDR gene mutations are displayed in **Supplementary Figure S4 and Supplementary Table S3**.

### The prognostic value of DDR gene mutations in validation cohorts

**Figure 2:**
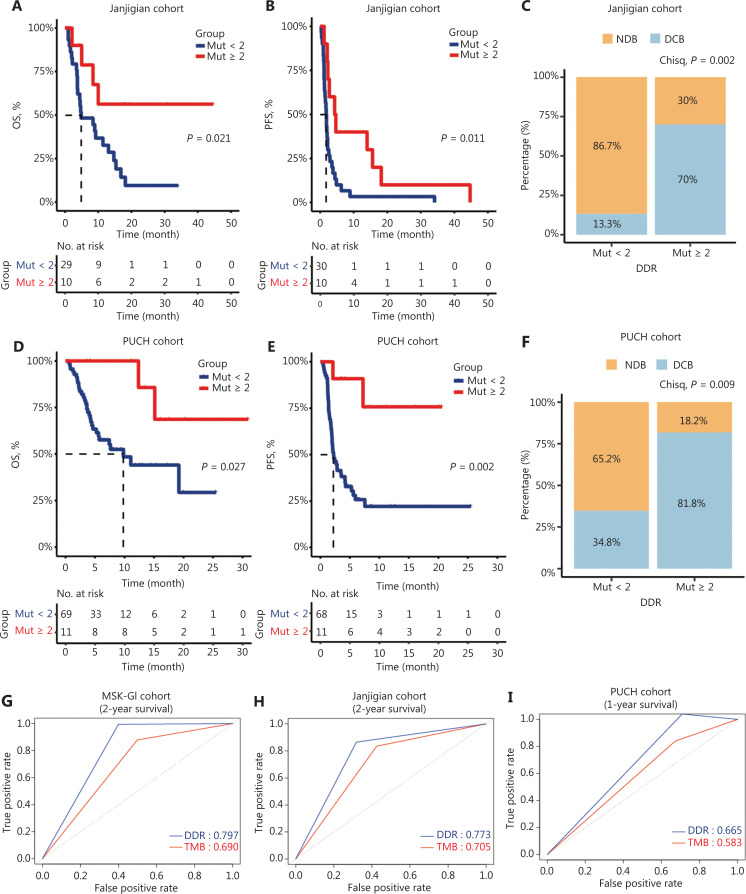
Clinical outcomes of patients with ≥ 2 and < 2 DNA damage response and repair (DDR) gene mutations in the validation cohorts. (A–B) Kaplan-Meier survival curves of the overall survival (OS) (A) and progression-free survival (PFS) (B) comparing ≥ 2 DDR gene mutations with < 2 DDR gene mutations in gastric cancer patients from the Janjigian cohort. (C) Percentage of DCB in patients with < 2 DDR gene mutations (*n* = 30) and ≥ 2 DDR gene mutations (*n* = 10) in the Janjigian cohort. (D–E) Kaplan-Meier curves for OS (D) and PFS (E) between ≥ 2 DDR gene mutations and < 2 DDR gene mutation subgroups in the Peking University Cancer Hospital & Institute (PUCH) cohort. (F) The DCB was compared between patients with ≥ 2 DDR gene mutations (*n* = 11) and those with < 2 DDR gene mutations (*n* = 69) in the PUCH cohort. (G–I) Time-dependent receiver operating characteristic curve analysis of the DDR gene mutation load and the tumor mutation burden in predicting survival outcomes in MSK-GI (2-year OS, G), Janjigian (2-year OS, H), and PUCH cohort (1-year OS, I). DCB, durable clinical benefit; NDB, no durable benefit.

To further investigate the prognostic significance of the DDR mutation status, 2 independent cohorts of ICI-treated GI cancer patients were analyzed. In the Janjigian cohort, 10 patients with ≥ 2 DDR gene mutations had a better OS and PFS than those with < 2 DDR gene mutations (median OS: NR *vs.* 4.8 months, *P* = 0.021; median PFS: 4.5 *vs.* 1.8 months, *P* = 0.011; **Figure 2A and 2B**). Moreover, patients with mutations in ≥ 2 DDR genes showed a considerably higher DCB than those with < 2 mutated DDR genes (70% *vs.* 13.3%, *P* = 0.002; **Figure 2C**). Similarly, in our PUCH cohort, patients with ≥ 2 DDR gene mutations had improved survival outcomes than patients with < 2 DDR gene mutations (median OS: NR *vs.* 9.8 months, *P* = 0.027; median PFS: NR *vs.* 2.2 months, *P* = 0.002; **Figure 2D and 2E**). Furthermore, patients with ≥ 2 DDR gene mutations demonstrated a significantly higher DCB than those with < 2 mutated DDR genes (81.8% *vs.* 34.8%, *P* = 0.009; **Figure 2F**).

Our univariant and multivariant Cox analyses confirmed that DDR gene mutations and the TMB were independent prognostic factors across 3 cohorts (**Table 2**). To compare the predictive power of these biomarkers, we used time-dependent ROC curve analysis. Our data revealed that DDR gene mutations indicated the higher area under the curve value than the TMB in all 3 cohorts (MSK-GI cohort: 0.797 *vs.* 0.690; Janjigian cohort: 0.773 *vs.* 0.705; PUCH cohort: 0.665 *vs.* 0.583; **Figure 2G–2I**). However, PD-L1 expression displayed a moderate predictive power in the Janjigian and PUCH cohorts (**Supplementary Figure S5**).

**Table 2:** Univariate and multivariate Cox analysis for overall survival in 3 cohorts

Variables	Univariate analysis			Multivariate analysis		
	HR	95%CI	*P*	HR	95%CI	*P*
MSK GI cohort^a^						
DDR mutations (≥ 2 *vs.* < 2)	0.32	0.17–0.58	< 0.001	0.32	0.17–0.60	< 0.001
TMB (high *vs.* low)	0.38	0.22–0.66	< 0.001	0.41	0.24–0.73	< 0.002
Janjigian cohort^b^						
DDR mutations (≥ 2 *vs.* < 2)	0.30	0.10–0.88	0.029	0.22	0.07–0.71	0.012
TMB (high *vs.* low)	0.38	0.14–1.00	0.053	0.21	0.07–0.64	0.006
PD-L1 (positive *vs.* negative)	0.24	0.08–0.77	0.016	0.09	0.02–0.51	0.006
PUCH cohort^c^						
DDR mutations (≥ 2 *vs.* < 2)	0.22	0.05–0.95	0.042	0.14	0.03–0.64	0.011
TMB (high *vs.* low)	0.35	0.14–0.89	0.027	0.23	0.09–0.63	0.004
PD-L1 (positive *vs.* negative)	0.85	0.37–1.90	0.696	0.90	0.39–2.10	0.809

^a^The multivariate analysis in the MSK-GI cohort was adjusted for age, gender, cancer type, drug, and metastasis. ^b^The multivariate analysis in the Janjigian cohort was adjusted for age, gender, HER2 and liver metastasis. ^c^The multivariate analysis in the PUCH cohort was adjusted for age, gender, immunotherapy and primary tumor.

To determine whether the DDR gene mutation load could be considered as a biomarker for nonimmunotherapy treatment, we analyzed the WES and clinical data of GI cancer in TCGA dataset and PUCH-ACT cohort containing 92 gastric cancer patients^26^. Intriguingly, the DDR gene mutation was not significantly associated with improved OS in either TCGA or the PUCH-ACT cohort (**Supplementary Figure S6**).

### Correlation between DDR mutation status and the TME

**Figure 3:**
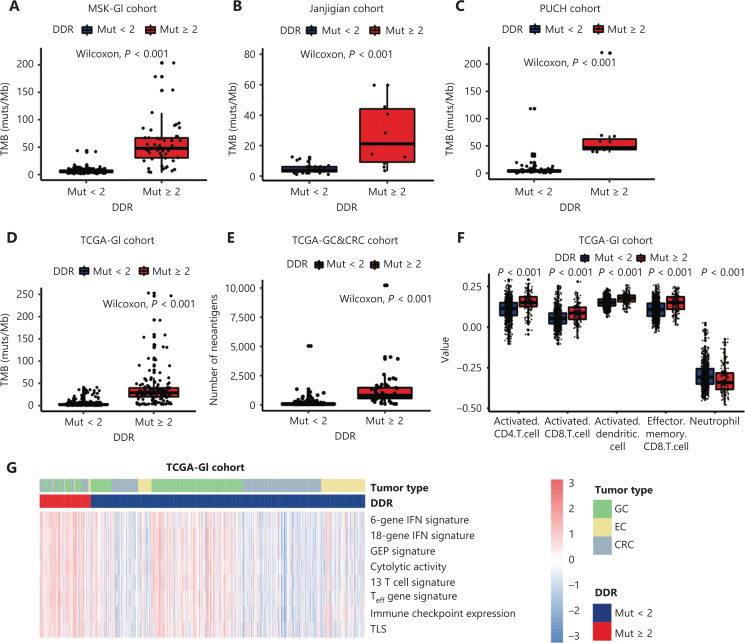
Correlation between DNA damage response and repair (DDR) gene mutations and the tumor microenvironment. (A–D) The tumor mutation burden between patients with ≥ 2 DDR gene mutations and < 2 DDR gene mutations in the MSK-GI cohort (A), the Janjigian cohort (B), the PUCH cohort (C) and TCGA-GI cancer cohort (D). (E) The number of neoantigens comparing patients with ≥ 2 DDR gene mutations and < 2 from TCGA database. (F) Comparison of the immune cell infiltration between the ≥ 2 and < 2 DDR gene mutation subgroups in the analysis of GI cancers. (G) Heat map showing the differentially expressed immune signatures between the subgroup of patients with ≥ 2 DDR gene mutations and the subgroup with < 2 DDR gene mutations in TCGA. GI, gastrointestinal; TMB, tumor mutation burden; GEP, gene expression profile; T_eff_, effector T cell; TLS, tertiary lymphoid structures; EC, esophageal cancer; GC, gastric cancer; CRC, colorectal cancer.

To further identify the mechanisms involving the impact of DDR gene mutations on clinical outcomes of ICI-treated GI cancer patients, we next investigated the influence of DDR gene mutations on the TME. First, a significantly increased level of TMB was observed in patients with ≥ 2 DDR gene mutations compared to < 2 DDR gene mutations in the MSK-GI, Janjigian, PUCH, and TCGA-GI cohorts (*P* < 0.001, **Figure 3A–3D**). The DDR gene mutation load was positively correlated with the TMB in these 3 cohorts (**Supplementary Figure S7**). Furthermore, gastric cancer and colorectal cancer patients from TCGA with ≥ 2 DDR gene mutations had higher neoantigen levels than those with fewer mutations (*P* < 0.001, **Figure 3E**).

We next focused on the relationship between DDR gene mutation and immune cell infiltration using ssGSEA methodology. Patients with ≥ 2 DDR gene mutations had greater infiltration of effective immune cells, such as activated dendritic cells (DCs), CD4+ T cells, and CD8+ T cells, but had fewer immunosuppressive cells than patients with < 2 DDR gene mutations (**Figure 3F**, *P* < 0.001). Moreover, tumors with ≥ 2 DDR gene mutations exhibited significant enrichment in immune-related signatures (**Figure 3G and Supplementary Figure S8**, *P* < 0.001). We also explored the influence of mutations in different DDR pathways on the immune environment in TCGA dataset, which showed that patients with any DDR pathway mutations had a favorable immune infiltration and enhanced immune-related signatures (**Supplementary Figure S9**). Together, these results indicated that the presence of DDR gene mutations could predict T cell inflammation phenotypes in GI cancer.

## Discussion

In this multicohort study, we investigated the prognostic role of DDR gene mutations in GI cancer patients receiving ICIs. Moreover, our study showed that the presence of ≥ 2 DDR gene mutations induced a distinct immune-activated microenvironment with an increased infiltration of immune cells, TMB, and neoantigens.

To date, several studies have been conducted to determine the efficacy of immunotherapy in GI cancer patients, but only approximately 10%–30% of patients benefit from ICIs^30,31^. Although the U.S. Food and Drug Administration has also approved mismatch repair deficient (dMMR)/MSI-H status as a biomarker for pembrolizumab utilization in treating solid tumors, less than 5% of advanced GI cancer patients harbor this marker^7,32^. TMB remains a controversial biomarker in GI cancer^33,34^, encountering several issues, including the lack of consensus regarding the cutoff point and the distinct immunologic impact of each gene mutation^35,36^. Emerging studies have demonstrated that mutations in some specific pathways or genes may exert positive or negative effects on the outcomes of ICI treatment^37–41^. Among these aberrations, the DDR gene mutation is a critical parameter predicting immunogenicity, and has been established as a promising biomarker of immunotherapy in urothelial cancer and NSCLC^22,23^, while its predictive value in GI cancer remains unclear.

Notably, DDR gene alterations are relatively common, occurring in approximately 17% of GI carcinomas, which is higher than the prevalence of dMMR/MSI-H^42^. To our knowledge, this study is the first to show a correlation between DDR gene mutations and clinical benefits from ICIs in GI cancer patients. We first divided patients into two subgroups by DDR mutation status. DDR gene mutation positivity (≥ 1) generally estimated the clinical outcomes and prognoses with different predictive values in various tumors (**Supplementary Figure S1**). However, with this kind of grouping, the prognostic value of DDR gene mutation (cutoff = 1) was not evident in esophagogastric cancer patients. We speculated that only 1 DDR gene mutation was not sufficient to contribute to a favorable immune environment, or to confer a survival benefit to GI cancer patients treated with ICIs. In fact, an increasing number of DDR gene alterations have shown a trend toward increased TMB and response to ICIs in urothelial carcinoma and NSCLC^21,22^. We therefore adjusted the cutoff points of the DDR gene mutation number, and found that setting the DDR gene mutations = 2 stratified GI cancer patients with distinct prognoses across 3 cohorts (**Figure 1A–1C, Figure 2A–2F, Supplementary Figure S1 and S2**)^43^. Importantly, our results further suggested that DDR gene mutation load was an independent prognostic factor (**Table 2**), showing a powerful predictive value for survival of GI cancer patients (**Figure 2G–2I**). In addition, the relationship between DDR gene mutation load and outcomes in GI cancer patients might vary by treatment, with the prognostic value of DDR gene mutation mainly observed in patients treated by ICIs.

Mechanistically, extensive efforts have been made to understand how DDR gene mutations influence the TME. Tumors with somatic DDR gene mutations manifest as an immune activation profile with increased TMB and neoantigens in multiple cancer types^43,44^, including GI cancer, as shown in the present study. In addition, DDR gene deficiency has been related to high levels of the chemokines, CXCL10 and CCL5, which are important for immune cell trafficking^17^. These findings suggested that DDR gene alterations in cancer cells generate a proinflammatory environment. Consistent with previous studies, we showed that tumors with ≥ 2 DDR gene mutations exhibited an enrichment of DCs, CD4+ T cells, CD8+ T cells, and immune-related signatures (**Figure 3F and 3G**). Among these enhanced signatures, the T cell inflammation gene expression profile (GEP) and tertiary lymphoid structures (TLS) were previously shown to predict benefits with ICI treatment^45,46^. Taken together, these results indicated that DDR gene mutations could predict T cell infiltration in GI cancer and hence predict the immunotherapeutic benefits.

There were several limitations in our study. (1) Due to the retrospective nature of this analysis, our study requires further validation in prospective clinical trials. (2) Our DDR gene profile may not have included all DDR-related genes. However, the 34 genes used in our study were the most representative genes reflective of the DDR system, which has been verified in previous studies^21,23^.

## Conclusions

In summary, our study elucidates that the presence of ≥ 2 DDR gene mutations correlated with improved survival in ICI-treated patients, and increased levels of T cell inflammation. However, further prospective studies are needed to validate this observation across multiple GI cancer types.
