# Supplementary material for: Alterations in DNA damage response and repair genes as potential biomarkers for immune checkpoint blockade in gastrointestinal cancer
Source: Cancer Biol Med. 2021 Sep 28;19(8):1139–49. doi: 10.20892/j.issn.2095-3941.2020.0708 (PMC9425187; doi:10.20892/j.issn.2095-3941.2020.0708)
Supplement: Supplementary file 1 [file cbm-19-1139-s001.pdf]

Supplementary materials

A **Hazard ratio of MSK pan-cancer cohort**

Tumor type	DDR negative (%)	DDR positive (%)	HR (95%CI)	*P* value
Bladder cancer	111 (52.6%)	100 (47.4%)	0.66 (0.43–1)	0.049
Breast cancer	33 (80.5%)	8 (19.5%)	0.3 (0.09–1.02)	0.053
Colorectal cancer	59 (54.1%)	50 (45.9%)	0.38 (0.19–0.73)	0.0036
Esophagogastric cancer	79 (66.9%)	39 (33.1%)	0.8 (0.44–1.43)	0.45
Glioma	98 (84.5%)	18 (15.5%)	1.08 (0.59–1.99)	0.81
Head and neck cancer	89 (69%)	40 (31%)	0.81 (0.49–1.36)	0.43
Melanoma	173 (55.3%)	140 (44.7%)	0.55 (0.38–0.8)	0.0017
Non-small cell lung cancer	215 (62.5%)	129 (37.5%)	0.75 (0.57–1)	0.051
Renal cell carcinoma	126 (88.1%)	17 (11.9%)	0.87 (0.37–2.03)	0.75

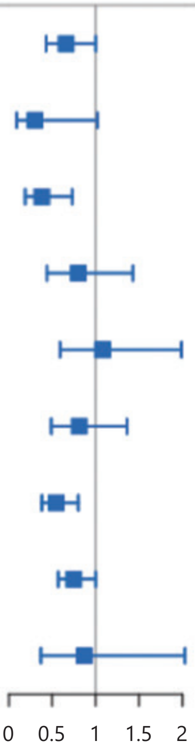

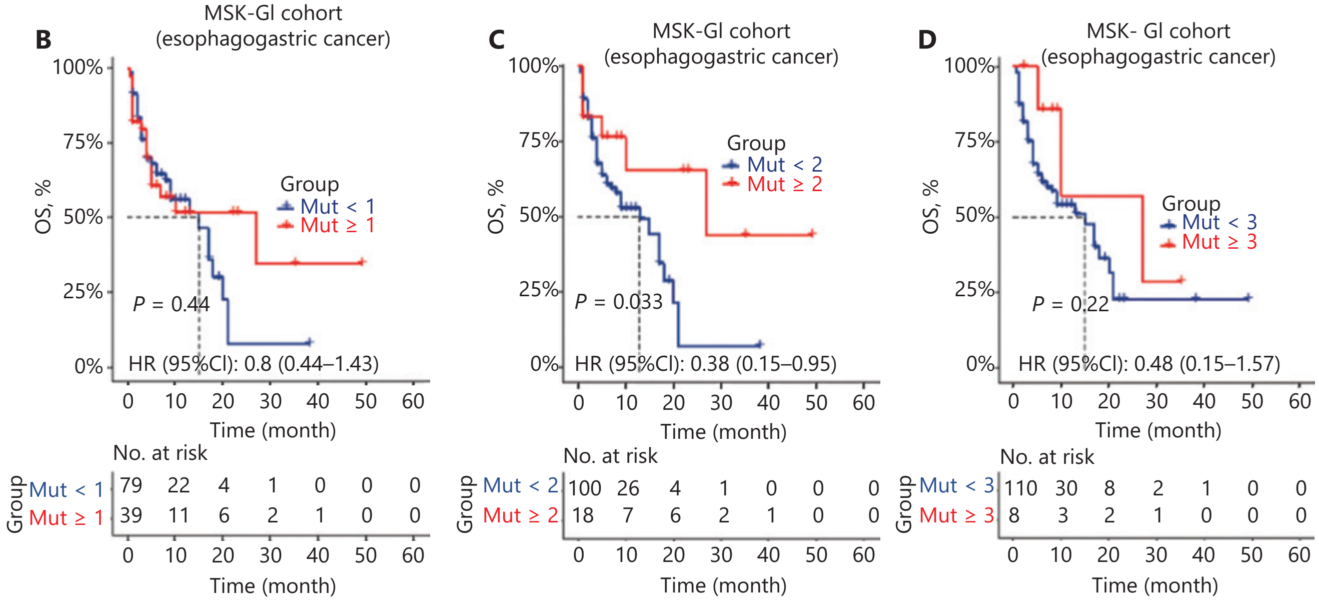

Figure S1 Continued

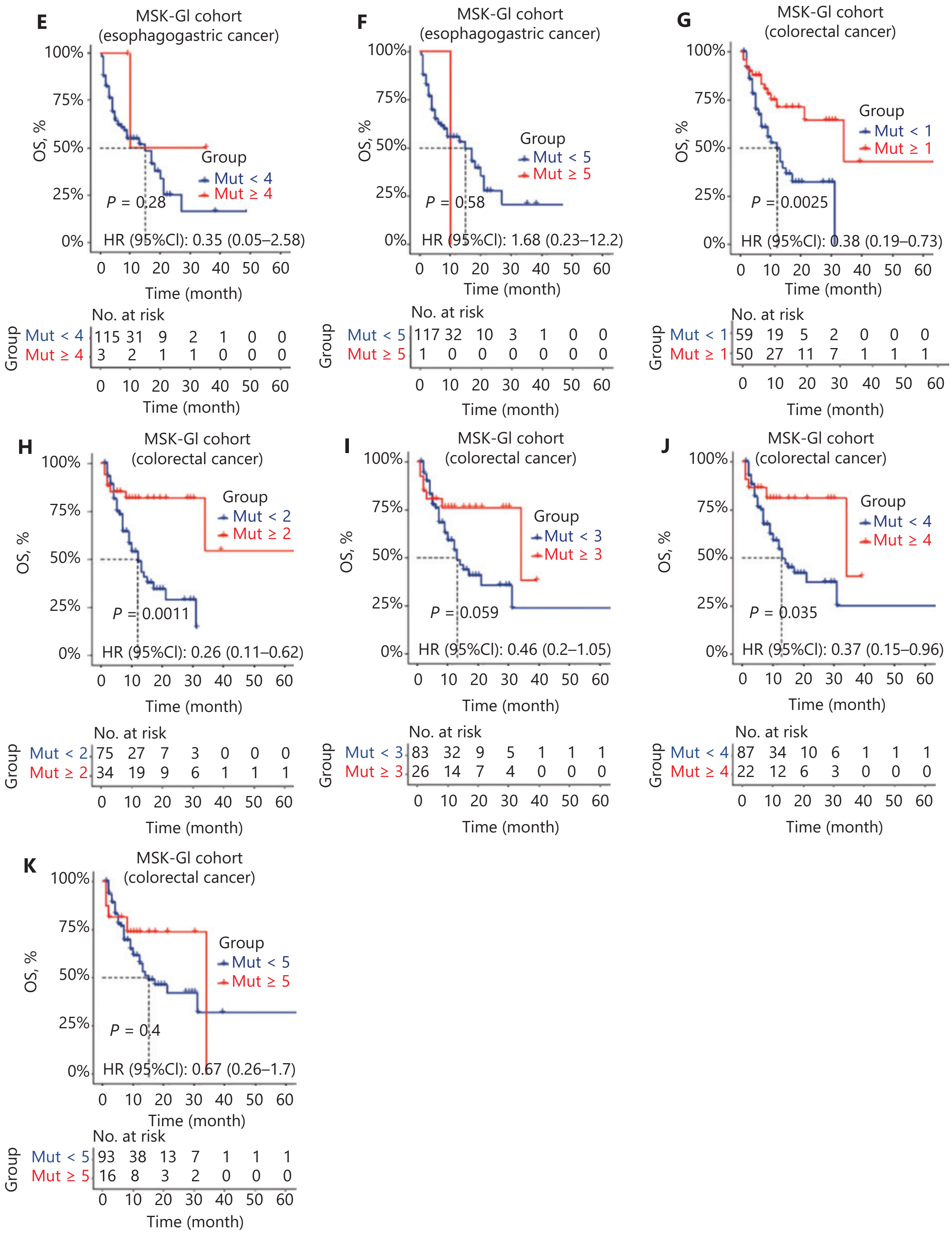

Figure S1 Prognostic role of DDR gene mutations in gastrointestinal cancer patients in the Memorial Sloan Kettering (MSK) cohort. (A) Forest plot of the hazard ratios (univariate analysis) describing the association between the DDR gene mutation load (≥ 1 *vs.* < 1) and overall survival in pan-cancer from the MSK cohort. (B–K) Kaplan-Meier curve for evaluating the overall survival of patients by different DDR gene mutation load in the MSK-GI cohort. DDR, DNA damage response and repair; mut, mutation.

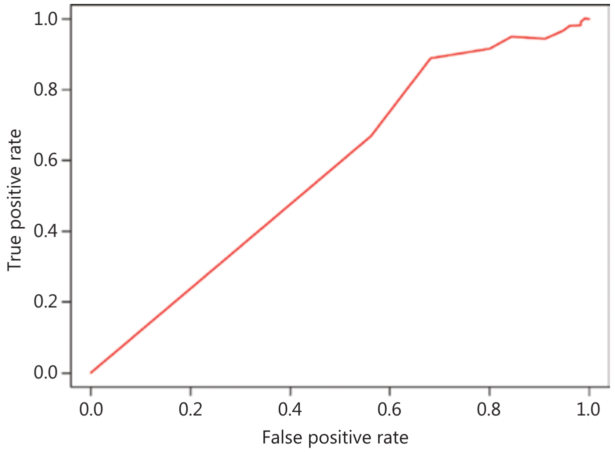

Figure S2 Time-dependent receiver operating characteristic (ROC) curve analysis of DNA damage response and repair gene mutation load as a continuous variable in predicting the overall survival (2-year survival) in the Memorial Sloan Kettering-gastrointestinal cancer cohort.

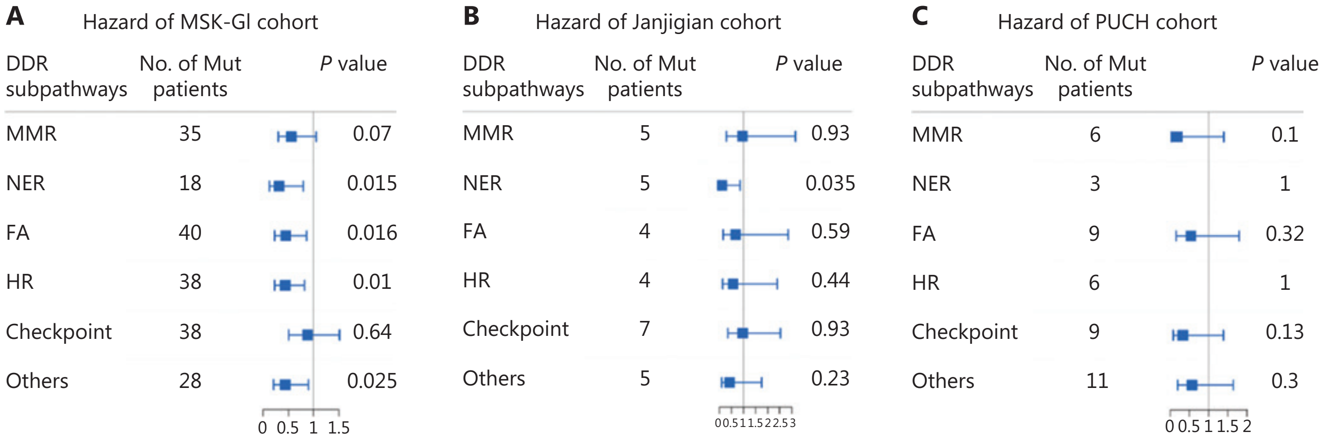

Figure S3 Forest plot of hazard ratios (univariate analysis) describing the association between each DNA damage response and repair pathway mutation and overall survival in the MSK GI cohort (A), the Janjigian cohort (B), and the PUCH cohort (C). FA, Fanconi anemia; HR, homologous recombination; MMR, mismatch repair; NER, nucleotide excision repair.

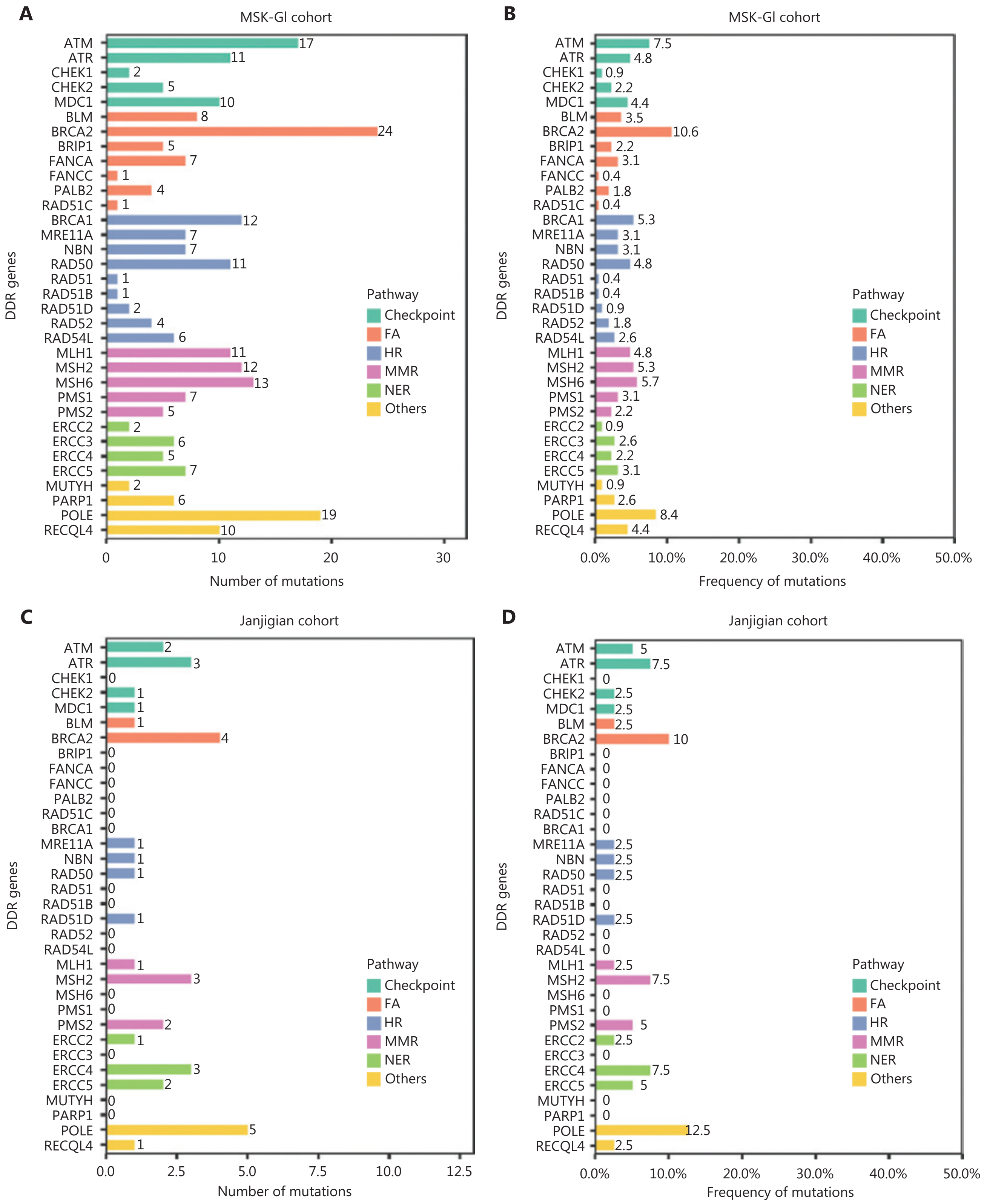

Figure S4 Continued

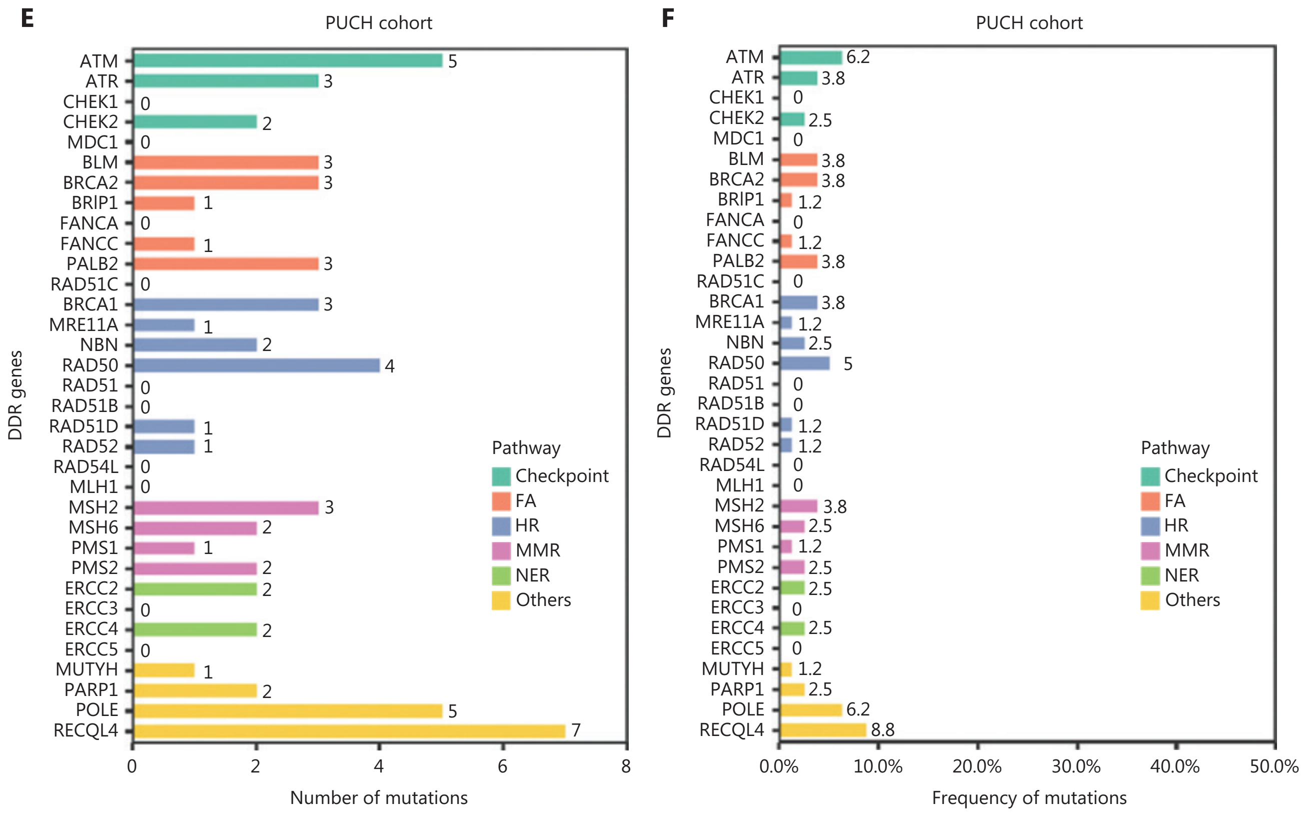

Figure S4 DDR gene mutations by pathways and mechanisms in 3 cohorts. (A–F) Number and frequency of DDR gene mutations in the MSK GI cohort (A and B), the Janjigian cohort (C and D), and the PUCH cohort (E and F). DDR, DNA damage response and repair; FA, Fanconi anemia; HR, homologous recombination; MMR, mismatch repair; NER, nucleotide excision repair.

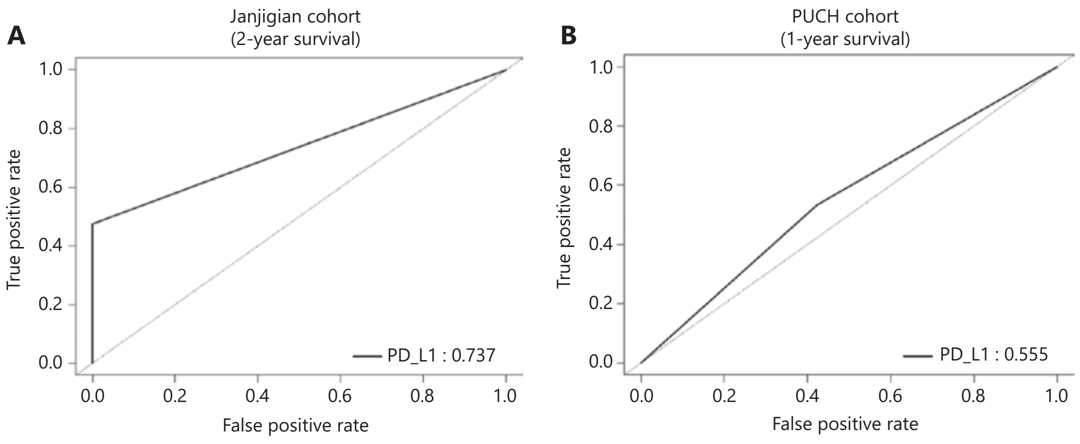

Figure S5 Time-dependent receiver operating characteristic curves to evaluate the sensitivity and specificity of the PD-L1 in predicting overall survival in the Janjigian cohort (A) and PUCH cohort (B).

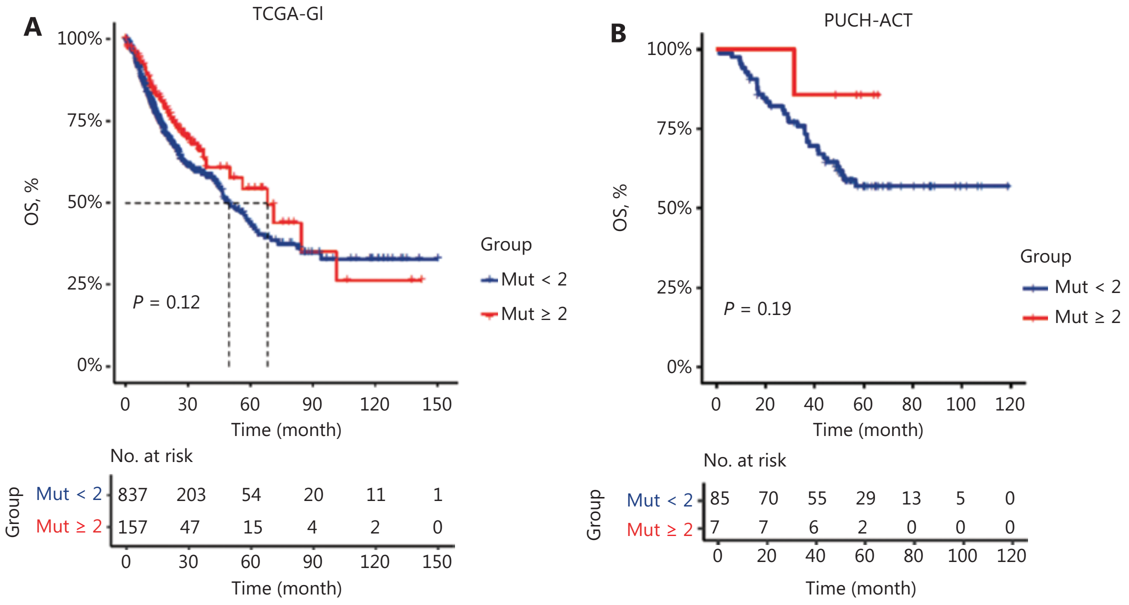

Figure S6 Kaplan-Meier analysis of patients harboring ≥ 2 and < 2 DNA damage response and repair gene mutations in non-immunotherapy cohorts. (A) TCGA-gastrointestinal cohort; (B) the PUCH-ACT cohort.

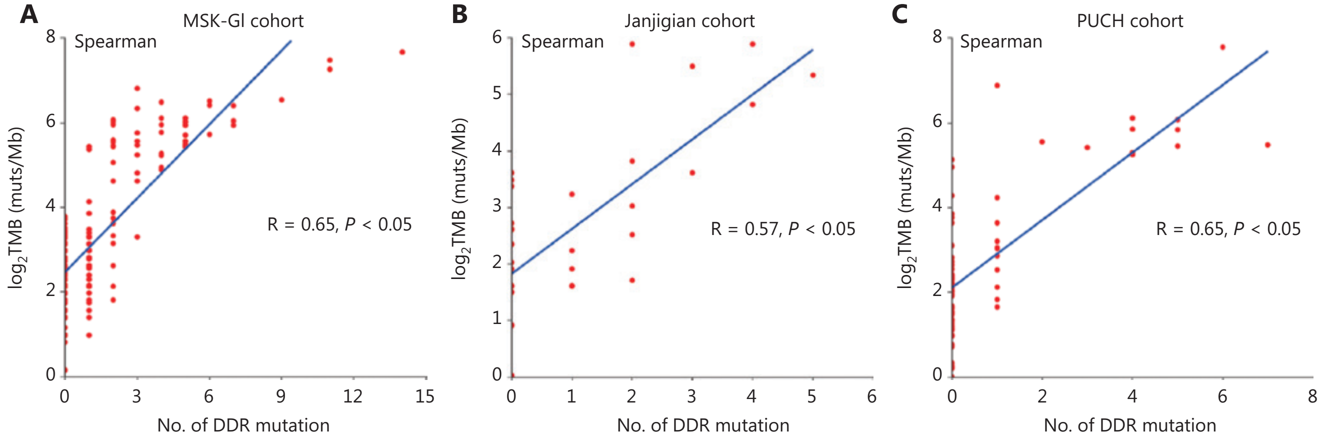

Figure S7 The correlation between the DNA damage response and repair gene mutation load and the tumor mutation burden in the MSK GI cohort (A), the Janjigian cohort (B), and the PUCH cohort (C).

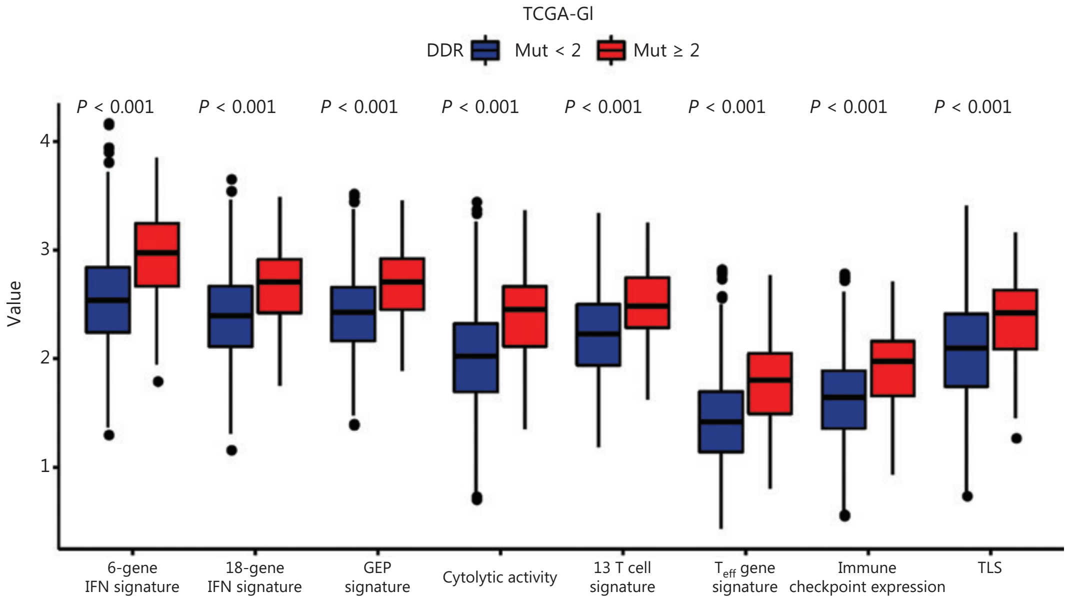

Figure S8 The association of DNA damage response and repair gene mutation with immune related signatures in TCGA-gastrointestinal cancer cohort. GEP, gene expression profile; T_{eff}, effector T cell; TLS, tertiary lymphoid structures.

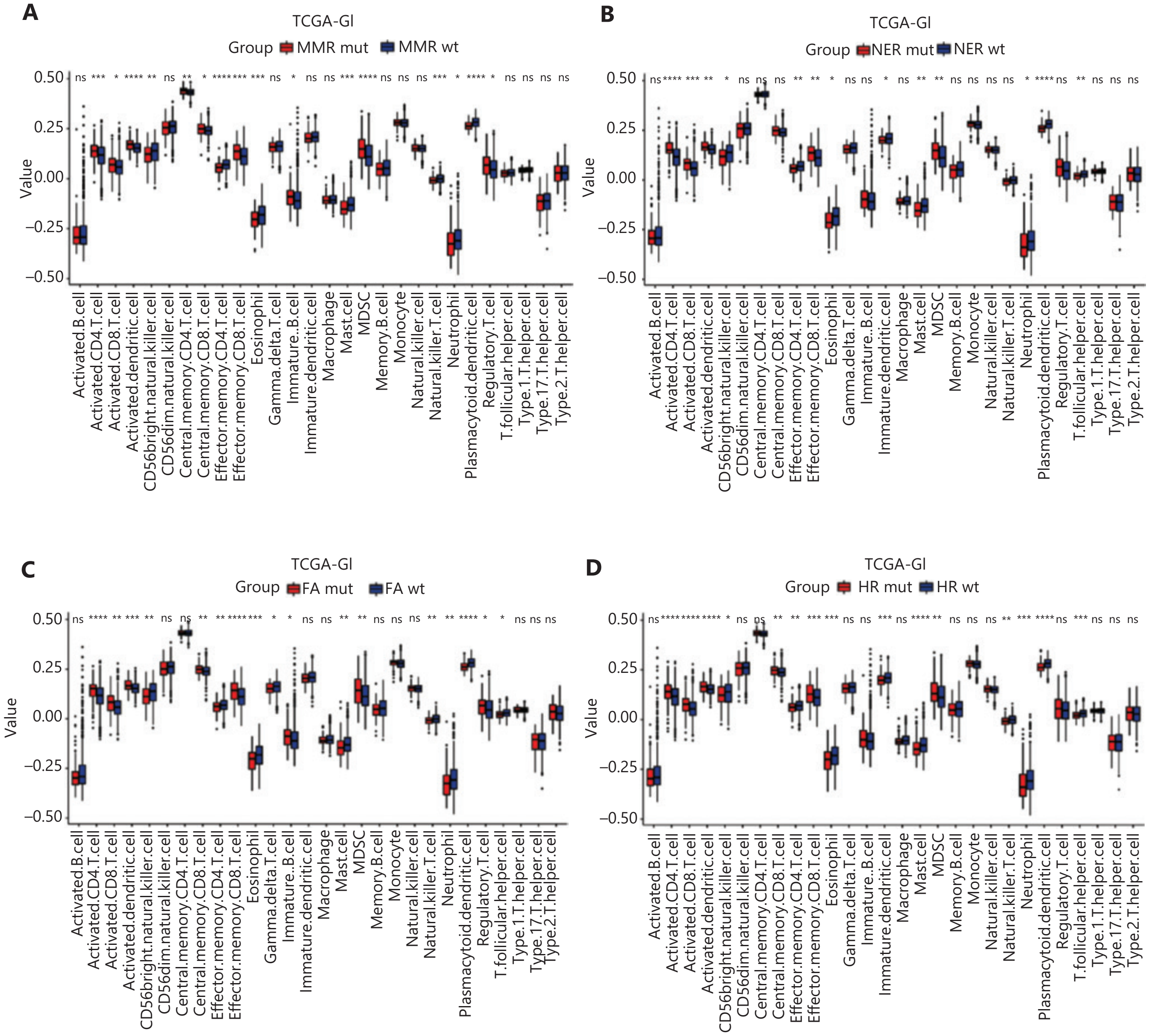

Figure S9 Continued

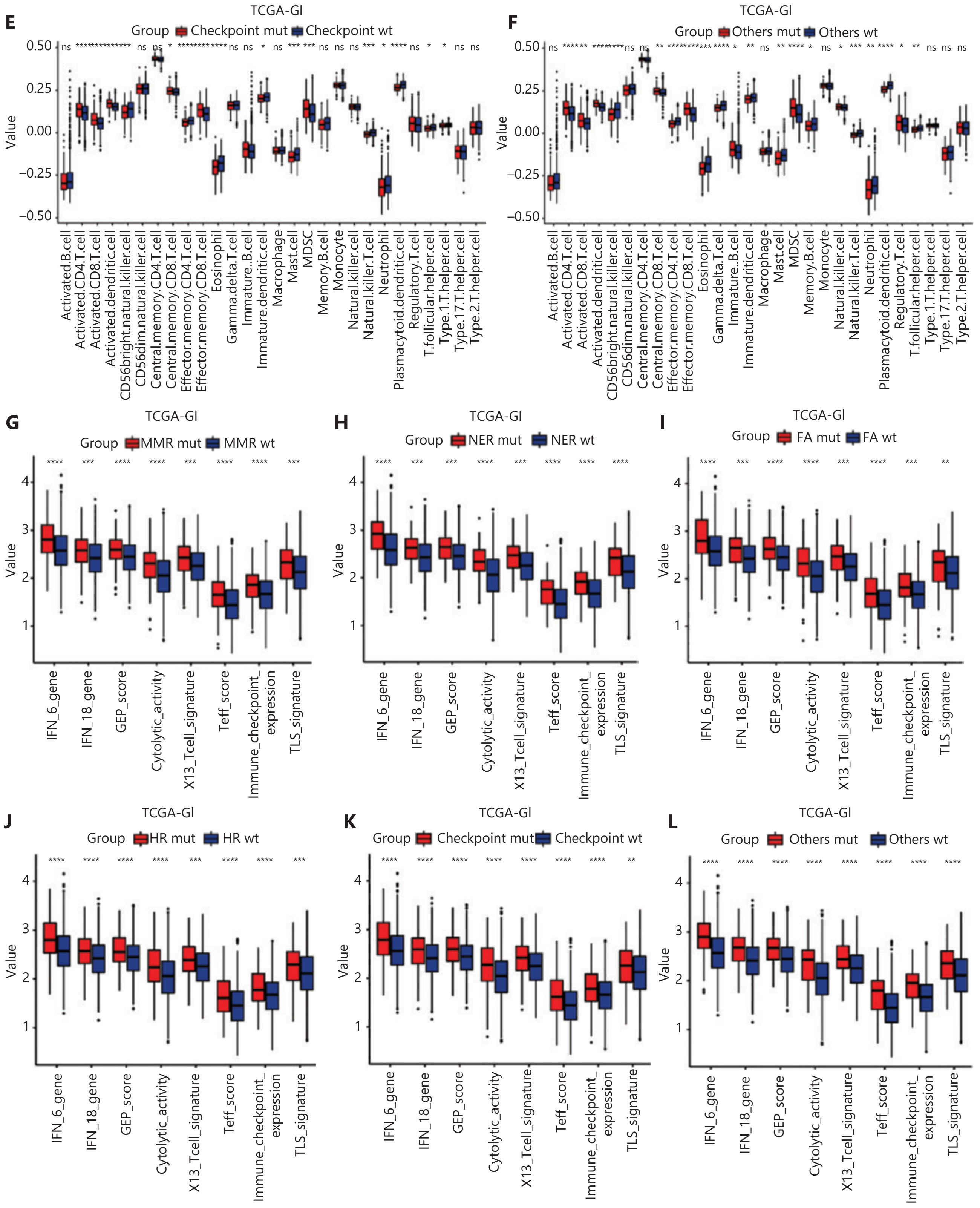

Figure S9 Comparison of the immune status between each DNA damage response and repair pathway mutation subgroup and corresponding wildtype subgroup in TCGA-gastrointestinal cohort. (A–F) Immune cell infiltration. (G–L) Immune-related signatures. FA, Fanconi anemia; HR, homologous recombination; MMR, mismatch repair; NER, nucleotide excision repair; mut, mutation; wt, wildtype.

Table S1 The DNA damage response and repair gene panel

MMR	NER	HR	FA	Checkpoint	Others
MLH1	ERCC2	BRCA1	BRCA2	ATM	POLE
MSH2	ERCC3	MRE11A	BRIP1	ATR	MUTYH
MSH6	ERCC4	NBN	FANCA	CHEK1	PARP1
PMS1	ERCC5	RAD50	FANCC	CHEK2	RECQL4
PMS2		RAD51	PALB2	MDC1	
		RAD51B	RAD51C		
		RAD51D	BLM		
		RAD52			
		RAD54L			

MMR, mismatch repair; NER, nucleotide excision repair; HR, homologous recombination; FA, fanconi anemia.

Table S2 Public-available gene signatures used in the study

Signature name	References
6-gene IFN signature	Ayers M et al, J Clin Invest. 2017;127:2930-2940
18-gene IFN signature	Ayers M et al, J Clin Invest. 2017;127:2930-2940
Gene expression profile	Cristescu R, et al, Science. 2018;362
Cytolytic activity	Rooney et al. Cell. 2015;160:48-61
13 T-cell signature	Spranger et al. Proc Natl Acad Sci U S A. 2016;113(48):E7759-E7768.
Teff gene signature	McDermott DF, Nat Med, 2018;24(6):749-757
Immune checkpoint expression	Sanchez A, Lancet Oncol. 2020;21(2):283-293
TLS	Finkin et al. Nat Immunol. 2015;16:1235-44

IFN, interferon; TLS, tertiary lymphoid structure; Teff, effector T cell.

Table S3 The mutation file of DNA damage response and repair genes in 3 cohorts

MSK-GI cohort		
Patient	Gene	Type of alterations
P-0000682-T01-IM3	RAD52	Missense_Mutation
P-0000682-T01-IM3	BRCA2	Missense_Mutation
P-0000793-T02-IM5	RAD51D	Missense_Mutation
P-0001260-T01-IM3	MDC1	Missense_Mutation
P-0001771-T01-IM3	POLE	Missense_Mutation
P-0001808-T01-IM3	PARP1	Missense_Mutation
P-0001808-T01-IM3	POLE	Missense_Mutation
P-0001808-T01-IM3	MSH6	Missense_Mutation
P-0001808-T01-IM3	ATR	Missense_Mutation
P-0001808-T01-IM3	FANCC	Missense_Mutation
P-0001808-T01-IM3	ATM	Missense_Mutation
P-0001808-T01-IM3	BRCA2	Missense_Mutation
P-0001808-T01-IM3	BLM	Missense_Mutation
P-0001808-T01-IM3	FANCA	Missense_Mutation
P-0001808-T01-IM3	BRCA1	Missense_Mutation
P-0001808-T01-IM3	ATR	Nonsense_Mutation
P-0001808-T01-IM3	RAD52	Nonsense_Mutation
P-0001808-T01-IM3	BRCA2	Nonsense_Mutation
P-0001867-T01-IM3	PALB2	Missense_Mutation
P-0002042-T01-IM3	ATM	Missense_Mutation
P-0002049-T01-IM3	MSH6	Frame_Shift_Ins
P-0002049-T01-IM3	POLE	Missense_Mutation
P-0002049-T01-IM3	ERCC5	Missense_Mutation
P-0002049-T01-IM3	FANCA	Missense_Mutation
P-0003432-T01-IM5	ATR	Missense_Mutation
P-0003521-T02-IM5	RAD51B	Missense_Mutation
P-0003650-T01-IM5	MSH2	Frame_Shift_Del
P-0003650-T01-IM5	BRCA2	Frame_Shift_Del
P-0003650-T01-IM5	NBN	Missense_Mutation
P-0003650-T01-IM5	POLE	Missense_Mutation
P-0003914-T01-IM3	PMS2	Missense_Mutation
P-0003914-T01-IM3	ATM	Splice_Site
P-0004011-T01-IM5	RAD54L	Missense_Mutation
P-0004011-T01-IM5	RECQL4	Missense_Mutation

Table S3 Continued

MSK-GI cohort		
Patient	Gene	Type of alterations
P-0004011-T01-IM5	POLE	Missense_Mutation
P-0004011-T01-IM5	FANCA	Missense_Mutation
P-0004011-T01-IM5	MSH2	Nonsense_Mutation
P-0004051-T01-IM5	MRE11A	Frame_Shift_Del
P-0004051-T01-IM5	ERCC5	Frame_Shift_Del
P-0004051-T01-IM5	BRCA1	Frame_Shift_Del
P-0004051-T01-IM5	PALB2	Missense_Mutation
P-0004362-T01-IM5	BRCA2	Frame_Shift_Del
P-0004362-T01-IM5	PMS1	Frame_Shift_Del
P-0004362-T01-IM5	RECQL4	Missense_Mutation
P-0004362-T01-IM5	ERCC3	Missense_Mutation
P-0004362-T01-IM5	ATR	Missense_Mutation
P-0004494-T01-IM5	ATM	Missense_Mutation
P-0004783-T01-IM5	MRE11A	Frame_Shift_Del
P-0004783-T01-IM5	ATR	Frame_Shift_Ins
P-0004783-T01-IM5	MSH2	Missense_Mutation
P-0004783-T01-IM5	FANCA	Missense_Mutation
P-0004783-T01-IM5	BRCA1	Missense_Mutation
P-0005132-T01-IM5	NBN	Missense_Mutation
P-0005151-T01-IM5	BRCA2	Frame_Shift_Del
P-0005151-T01-IM5	RAD50	Frame_Shift_Del
P-0005151-T01-IM5	BRCA1	Frame_Shift_Del
P-0005151-T01-IM5	RAD54L	Missense_Mutation
P-0005151-T01-IM5	PMS1	Missense_Mutation
P-0005151-T01-IM5	BRCA2	Missense_Mutation
P-0005151-T01-IM5	PALB2	Missense_Mutation
P-0005151-T01-IM5	RAD51D	Missense_Mutation
P-0005151-T01-IM5	BRCA1	Missense_Mutation
P-0005500-T01-IM5	BRCA2	Frame_Shift_Del
P-0005500-T01-IM5	MDC1	Missense_Mutation
P-0005762-T01-IM5	BRCA2	Missense_Mutation
P-0005823-T01-IM5	CHEK2	Frame_Shift_Del
P-0005823-T01-IM5	MSH2	In_Frame_Del
P-0005823-T01-IM5	MSH2	Missense_Mutation
P-0005823-T01-IM5	ERCC3	Missense_Mutation

Table S3 Continued

MSK-GI cohort		
Patient	Gene	Type of alterations
P-0005823-T01-IM5	RAD50	Missense_Mutation
P-0005823-T01-IM5	CHEK2	Missense_Mutation
P-0005823-T01-IM5	MSH2	Nonsense_Mutation
P-0005823-T01-IM5	RAD50	5′-UTR
P-0007531-T01-IM5	BRCA2	Frame_Shift_Del
P-0007531-T01-IM5	RECQL4	Missense_Mutation
P-0007531-T01-IM5	POLE	Missense_Mutation
P-0007995-T01-IM5	ERCC4	Frame_Shift_Del
P-0007995-T01-IM5	ATR	Missense_Mutation
P-0007995-T01-IM5	PMS2	Missense_Mutation
P-0007995-T01-IM5	MLH1	Splice_Site
P-0007997-T01-IM5	PMS2	Frame_Shift_Del
P-0007997-T01-IM5	MDC1	Missense_Mutation
P-0007997-T01-IM5	POLE	Missense_Mutation
P-0007997-T01-IM5	BRCA2	Missense_Mutation
P-0007997-T01-IM5	RAD51	Missense_Mutation
P-0008188-T01-IM5	MRE11A	Frame_Shift_Del
P-0008188-T01-IM5	ATM	Missense_Mutation
P-0008188-T01-IM5	POLE	Missense_Mutation
P-0008746-T01-IM5	MRE11A	Frame_Shift_Del
P-0008746-T01-IM5	RAD51C	Missense_Mutation
P-0009057-T01-IM5	BLM	Frame_Shift_Del
P-0009057-T01-IM5	MSH2	Missense_Mutation
P-0009057-T01-IM5	BRCA2	Missense_Mutation
P-0009057-T01-IM5	ERCC5	Missense_Mutation
P-0009057-T01-IM5	ERCC4	Missense_Mutation
P-0009066-T01-IM5	POLE	Nonsense_Mutation
P-0009563-T01-IM5	CHEK2	Missense_Mutation
P-0009563-T01-IM5	BRCA1	Missense_Mutation
P-0009830-T01-IM5	BRCA1	Frame_Shift_Del
P-0009830-T01-IM5	BRCA2	Frame_Shift_Ins
P-0009830-T01-IM5	MLH1	Missense_Mutation
P-0009830-T01-IM5	BRCA2	Missense_Mutation
P-0009918-T01-IM5	POLE	Missense_Mutation

Table S3 Continued

MSK-GI cohort		
Patient	Gene	Type of alterations
P-0009918-T01-IM5	ERCC2	Missense_Mutation
P-0010238-T01-IM5	ATM	Missense_Mutation
P-0010499-T01-IM5	BRCA2	Frame_Shift_Ins
P-0010499-T01-IM5	MSH6	Missense_Mutation
P-0010499-T01-IM5	ERCC5	Missense_Mutation
P-0010499-T01-IM5	ATR	Missense_Mutation
P-0010499-T01-IM5	ATM	Missense_Mutation
P-0010499-T01-IM5	PMS1	Missense_Mutation
P-0010499-T01-IM5	MLH1	Missense_Mutation
P-0010499-T01-IM5	RAD50	Missense_Mutation
P-0010499-T01-IM5	RECQL4	Missense_Mutation
P-0010499-T01-IM5	POLE	Missense_Mutation
P-0010499-T01-IM5	BRCA2	Missense_Mutation
P-0010499-T01-IM5	BLM	Missense_Mutation
P-0010499-T01-IM5	ATR	Nonsense_Mutation
P-0010499-T01-IM5	PMS1	Nonsense_Mutation
P-0010499-T01-IM5	BLM	Nonsense_Mutation
P-0010499-T01-IM5	ATR	Splice_Site
P-0010501-T01-IM5	MSH2	Missense_Mutation
P-0010581-T01-IM5	RECQL4	Missense_Mutation
P-0010581-T01-IM5	CHEK1	Missense_Mutation
P-0010587-T01-IM5	MSH6	Frame_Shift_Ins
P-0010587-T01-IM5	MDC1	Missense_Mutation
P-0010587-T01-IM5	NBN	Missense_Mutation
P-0010587-T01-IM5	POLE	Missense_Mutation
P-0010587-T01-IM5	BLM	Missense_Mutation
P-0010587-T01-IM5	MSH2	Nonsense_Mutation
P-0010587-T01-IM5	RAD50	Splice_Site
P-0011068-T01-IM5	ATM	Frame_Shift_Del
P-0011068-T01-IM5	MLH1	Missense_Mutation
P-0011071-T01-IM5	NBN	Frame_Shift_Del
P-0011071-T01-IM5	MSH6	Frame_Shift_Del
P-0011071-T01-IM5	MSH6	Missense_Mutation
P-0011071-T01-IM5	MLH1	Nonsense_Mutation

Table S3 Continued

MSK-GI cohort		
Patient	Gene	Type of alterations
P-0011144-T01-IM5	ATR	Missense_Mutation
P-0011357-T01-IM5	MUTYH	Missense_Mutation
P-0011357-T01-IM5	RAD54L	Missense_Mutation
P-0011357-T01-IM5	MSH6	Missense_Mutation
P-0011357-T01-IM5	ERCC3	Missense_Mutation
P-0011357-T01-IM5	PMS1	Missense_Mutation
P-0011357-T01-IM5	ATR	Missense_Mutation
P-0011357-T01-IM5	NBN	Missense_Mutation
P-0011357-T01-IM5	ATM	Missense_Mutation
P-0011357-T01-IM5	POLE	Missense_Mutation
P-0011357-T01-IM5	BRCA2	Missense_Mutation
P-0011357-T01-IM5	ERCC5	Missense_Mutation
P-0011357-T01-IM5	BLM	Missense_Mutation
P-0011357-T01-IM5	BRIP1	Missense_Mutation
P-0011357-T01-IM5	MSH6	Nonsense_Mutation
P-0011357-T01-IM5	ATR	Nonsense_Mutation
P-0011357-T01-IM5	RAD50	Nonsense_Mutation
P-0011357-T01-IM5	BRCA2	Nonsense_Mutation
P-0012115-T01-IM5	MLH1	Frame_Shift_Del
P-0012115-T01-IM5	BRIP1	Frame_Shift_Ins
P-0012115-T01-IM5	RAD54L	Missense_Mutation
P-0012249-T01-IM5	BRCA2	Frame_Shift_Del
P-0012249-T01-IM5	BRCA2	In_Frame_Del
P-0012249-T01-IM5	MRE11A	Missense_Mutation
P-0012249-T01-IM5	FANCA	Missense_Mutation
P-0012249-T01-IM5	ATM	Nonsense_Mutation
P-0012281-T01-IM5	ATM	Missense_Mutation
P-0012728-T01-IM5	ERCC4	Missense_Mutation
P-0013106-T01-IM5	ATR	Missense_Mutation
P-0013106-T01-IM5	ERCC4	Missense_Mutation
P-0013106-T01-IM5	ATR	Nonsense_Mutation
P-0013462-T01-IM5	RECQL4	Frame_Shift_Del
P-0013462-T01-IM5	RECQL4	Missense_Mutation
P-0014565-T01-IM6	BRCA2	Missense_Mutation

Table S3 Continued

MSK-GI cohort		
Patient	Gene	Type of alterations
P-0015237-T01-IM6	RAD50	Frame_Shift_Del
P-0015237-T01-IM6	MSH6	Frame_Shift_Del
P-0015237-T01-IM6	POLE	Frame_Shift_Del
P-0015237-T01-IM6	PARP1	Missense_Mutation
P-0015237-T01-IM6	ERCC5	Missense_Mutation
P-0015237-T01-IM6	MDC1	Missense_Mutation
P-0015331-T01-IM6	MSH6	Missense_Mutation
P-0015851-T01-IM6	NBN	Frame_Shift_Del
P-0015851-T01-IM6	PARP1	Missense_Mutation
P-0015851-T01-IM6	MSH2	Missense_Mutation
P-0015851-T01-IM6	MLH1	Splice_Site
P-0016181-T01-IM6	BRCA2	Frame_Shift_Del
P-0016181-T01-IM6	POLE	Missense_Mutation
P-0016233-T01-IM6	BRCA2	Missense_Mutation
P-0016311-T01-IM6	BRCA2	Missense_Mutation
P-0016311-T01-IM6	RAD50	Missense_Mutation
P-0016314-T01-IM6	MLH1	Missense_Mutation
P-0016314-T01-IM6	PMS2	Missense_Mutation
P-0016314-T01-IM6	RAD50	Splice_Site
P-0016522-T01-IM6	BRCA2	Frame_Shift_Del
P-0016522-T01-IM6	PMS1	Frame_Shift_Del
P-0016522-T01-IM6	RECQL4	Missense_Mutation
P-0016522-T01-IM6	MRE11A	Missense_Mutation
P-0016522-T01-IM6	BLM	Missense_Mutation
P-0017370-T01-IM6	ATM	Missense_Mutation
P-0017370-T01-IM6	RAD52	Missense_Mutation
P-0017370-T01-IM6	ATR	Missense_Mutation
P-0017697-T01-IM6	RAD52	Missense_Mutation
P-0017697-T01-IM6	ATM	Missense_Mutation
P-0017697-T01-IM6	CHEK2	Missense_Mutation
P-0017697-T01-IM6	MSH2	Missense_Mutation
P-0017697-T01-IM6	ERCC3	Missense_Mutation
P-0017697-T01-IM6	PMS2	Missense_Mutation
P-0017766-T01-IM6	ERCC4	Missense_Mutation

Table S3 Continued

MSK-GI cohort		
Patient	Gene	Type of alterations
P-0017986-T01-IM6	MSH6	Frame_Shift_Ins
P-0017986-T01-IM6	MSH2	Missense_Mutation
P-0017986-T01-IM6	BRCA1	Missense_Mutation
P-0017986-T01-IM6	MDC1	Missense_Mutation
P-0017986-T01-IM6	RECQL4	Missense_Mutation
P-0017986-T01-IM6	CHEK2	Nonsense_Mutation
P-0017986-T01-IM6	MUTYH	Splice_Region
P-0018143-T01-IM6	MDC1	Missense_Mutation
P-0018371-T01-IM6	CHEK2	In_Frame_Del
P-0018464-T01-IM6	POLE	Missense_Mutation
P-0018464-T01-IM6	ERCC5	Missense_Mutation
P-0018800-T01-IM6	BRCA2	Missense_Mutation
P-0018927-T01-IM6	PMS1	Missense_Mutation
P-0019271-T01-IM6	RECQL4	Missense_Mutation
P-0019272-T01-IM6	BRIP1	Missense_Mutation
P-0019351-T01-IM6	BRCA1	Missense_Mutation
P-0019428-T01-IM6	MSH6	Frame_Shift_Del
P-0019428-T01-IM6	BRCA1	Frame_Shift_Del
P-0019475-T01-IM6	ATM	Frame_Shift_Del
P-0019475-T01-IM6	MSH6	Frame_Shift_Del
P-0019475-T01-IM6	BRCA1	Frame_Shift_Del
P-0019475-T01-IM6	MSH2	Frame_Shift_Del
P-0019475-T01-IM6	BLM	Missense_Mutation
P-0019475-T01-IM6	PARP1	Missense_Mutation
P-0019475-T01-IM6	POLE	Missense_Mutation
P-0019475-T01-IM6	BRCA2	Missense_Mutation
P-0019475-T01-IM6	MLH1	Nonsense_Mutation
P-0019691-T01-IM6	NBN	Frame_Shift_Del
P-0019691-T01-IM6	POLE	Missense_Mutation
P-0019691-T01-IM6	RECQL4	Missense_Mutation
P-0019949-T01-IM6	BRCA1	Frame_Shift_Del
P-0019949-T01-IM6	RAD54L	Missense_Mutation
P-0019949-T01-IM6	BRIP1	Missense_Mutation
P-0019949-T01-IM6	ERCC3	Missense_Mutation

Table S3 Continued

MSK-GI cohort		
Patient	Gene	Type of alterations
P-0020169-T01-IM6	ATM	Nonsense_Mutation
P-0020252-T01-IM6	PALB2	Missense_Mutation
P-0020252-T01-IM6	BRCA2	Missense_Mutation
P-0020399-T01-IM6	MDC1	Frame_Shift_Del
P-0020399-T01-IM6	BRCA1	Frame_Shift_Ins
P-0020399-T01-IM6	RAD54L	Missense_Mutation
P-0020399-T01-IM6	POLE	Missense_Mutation
P-0020399-T01-IM6	MLH1	Splice_Site
P-0020903-T01-IM6	RAD50	Missense_Mutation
P-0021304-T01-IM6	PARP1	Frame_Shift_Del
P-0021304-T01-IM6	MSH6	Frame_Shift_Del
P-0021304-T01-IM6	PARP1	Missense_Mutation
P-0021304-T01-IM6	CHEK1	Missense_Mutation
P-0021304-T01-IM6	FANCA	Missense_Mutation
P-0021304-T01-IM6	ERCC3	Missense_Mutation
P-0021304-T01-IM6	MSH2	Nonsense_Mutation
P-0021993-T01-IM6	ATR	Missense_Mutation
P-0021993-T01-IM6	MDC1	Missense_Mutation
P-0022688-T01-IM6	ATM	Nonsense_Mutation
P-0022788-T01-IM6	ATM	In_Frame_Del
P-0022977-T01-IM6	MLH1	Missense_Mutation
P-0022995-T01-IM6	BRCA2	Frame_Shift_Del
P-0023128-T01-IM6	MRE11A	Missense_Mutation
P-0023128-T01-IM6	BRCA2	Missense_Mutation
P-0023963-T01-IM6	ERCC2	Missense_Mutation
P-0023963-T01-IM6	FANCA	Missense_Mutation
P-0023963-T01-IM6	PMS1	Missense_Mutation
P-0024068-T01-IM6	BLM	Frame_Shift_Del
P-0024068-T01-IM6	MDC1	Missense_Mutation
P-0024205-T01-IM6	PARP1	Missense_Mutation
P-0024243-T01-IM6	BRIP1	Missense_Mutation
P-0024243-T01-IM6	RAD50	5′-UTR
P-0024824-T01-IM6	RAD50	Missense_Mutation
P-0025532-T01-IM6	MSH6	Frame_Shift_Ins

Table S3 Continued

Janjigian Cohort		
Patient	Gene	Type of Alterations
P-0000369-T01-IM3	CHEK2	Frame_Shift_Del
P-0000793-T02-IM5	RAD51D	Missense_Mutation
P-0001260-T01-IM3	MDC1	Missense_Mutation
P-0003432-T01-IM5	ATR	Missense_Mutation
P-0003650-T01-IM5	NBN	Missense_Mutation
P-0003650-T01-IM5	POLE	Missense_Mutation
P-0003650-T01-IM5	MSH2	Frame_Shift_Del
P-0003650-T01-IM5	BRCA2	Frame_Shift_Del
P-0003914-T01-IM3	PMS2	Missense_Mutation
P-0003914-T01-IM3	ATM	Splice_Site
P-0007531-T02-IM5	BRCA2	Frame_Shift_Del
P-0007531-T02-IM5	RECQL4	Missense_Mutation
P-0007531-T02-IM5	POLE	Missense_Mutation
P-0007995-T01-IM5	ATR	Missense_Mutation
P-0007995-T01-IM5	PMS2	Missense_Mutation
P-0007995-T01-IM5	MLH1	Splice_Region
P-0007995-T01-IM5	ERCC4	Frame_Shift_Del
P-0008188-T01-IM5	MRE11A	Frame_Shift_Del
P-0008188-T01-IM5	ATM	Missense_Mutation
P-0008188-T01-IM5	POLE	Missense_Mutation
P-0009057-T01-IM5	BLM	Frame_Shift_Del
P-0009057-T01-IM5	MSH2	Missense_Mutation
P-0009057-T01-IM5	BRCA2	Missense_Mutation
P-0009057-T01-IM5	ERCC5	Missense_Mutation
P-0009057-T01-IM5	ERCC4	Missense_Mutation
P-0009918-T01-IM5	POLE	Missense_Mutation
P-0009918-T01-IM5	ERCC2	Missense_Mutation
P-0010501-T01-IM5	MSH2	Missense_Mutation
P-0013106-T01-IM5	ATR	Nonsense_Mutation
P-0013106-T01-IM5	ATR	Missense_Mutation
P-0013106-T01-IM5	ERCC4	Missense_Mutation
P-0016311-T01-IM6	BRCA2	Missense_Mutation
P-0016311-T01-IM6	RAD50	Missense_Mutation
P-0018464-T01-IM6	POLE	Missense_Mutation
P-0018464-T01-IM6	ERCC5	Missense_Mutation

Table S3 Continued

PUCH Cohort		
Patient	Gene	Type of Alterations
P5	BRCA1	Frameshift
P5	PARP1	Missense
P5	PMS1	Frameshift
P5	ATR	Missense
P5	NBN	Frameshift
P26	ATM	Missense
P26	MRE11A	Frameshift
P26	ERCC4	Missense
P26	ERCC2	Missense
P26	ATR	Missense
P28	BRCA2	Frameshift
P28	PMS2	Frameshift
P28	POLE	Missense
P28	ATR	Missense
P28	RECQL4	Missense
P30	MSH2	Frameshift
P30	POLE	Missense
P30	POLE	Splice
P30	BRCA1	Missense
P30	RECQL4	Missense
P32	PALB2	Frameshift
P36	RAD50	Frameshift
P41	ATM	Frameshift
P42	MSH2	Nonsense
P42	RAD51D	Missense
P42	BRCA1	Missense
P48	ATM	Missense
P48	POLE	Missense
P48	RAD50	Frameshift
P48	RECQL4	Missense
P52	BRCA2	Frameshift
P52	MSH6	Frameshift
P52	BLM	Frameshift
P52	PALB2	Missense
P52	ERCC2	Missense

Table S3 Continued

PUCH Cohort		
Patient	Gene	Type of Alterations
P52	MSH2	Splice
P52	RECQL4	Missense
P72	POLE	Missense
P77	BLM	Nonsense
P87	RECQL4	Nonsense
P90	POLE	Missense
P90	BRCA2	Missense
P90	BLM	Nonsense
P90	ERCC4	Missense
P90	BRIP1	Missense
P90	RAD50	Missense
P93	CHEK2	Frameshift
P93	CHEK2	Missense
P100	ATM	Frameshift
P100	RECQL4	Missense
P105	MUTYH	Missense
P106	NBN	Frameshift
P110	RAD52	Nonsense
P110	PALB2	Missense
P110	RAD50	Frameshift
P110	FANCC	Missense
P114	PARP1	Missense
P114	MSH6	Frameshift
P114	PMS2	Missense
P114	RECQL4	Missense
P117	ATM	Missense
P122	CHEK2	Missense
